# Early rhythm control in patients with acute decompensated heart failure: results from the CYCLE cohort study

**DOI:** 10.1093/europace/euaf314

**Published:** 2025-12-04

**Authors:** Marvin Kriz, Caroline Kellner, Benedikt N Beer, Jonas Sundermeyer, Angela Dettling, Lisa Besch, Maria Luisa Benesch Vidal, Nina Becher, Gianluigi Savarese, Paulus Kirchhof, Stefan Blankenberg, Christina Magnussen, Benedikt Schrage, Peter Moritz Becher

**Affiliations:** Department of Cardiology, University Medical Center Hamburg-Eppendorf, Martinistrasse 52, 20251 Hamburg, Germany; German Center for Cardiovascular Research (DZHK), Partner Site Hamburg/Luebeck/Kiel, Germany; Department of Cardiology, University Medical Center Hamburg-Eppendorf, Martinistrasse 52, 20251 Hamburg, Germany; Center for Population Health Innovation (POINT), University Heart & Vascular Center Hamburg, University Medical Center Hamburg-Eppendorf, Hamburg, Germany; Department of Cardiology, University Medical Center Hamburg-Eppendorf, Martinistrasse 52, 20251 Hamburg, Germany; German Center for Cardiovascular Research (DZHK), Partner Site Hamburg/Luebeck/Kiel, Germany; Department of Clinical Science and Education, Södersjukhuset, Karolinska Institutet, Sjukhusbacken 10, 11883 Stockholm, Sweden; Department of Cardiology, University Medical Center Hamburg-Eppendorf, Martinistrasse 52, 20251 Hamburg, Germany; German Center for Cardiovascular Research (DZHK), Partner Site Hamburg/Luebeck/Kiel, Germany; The Cardiovascular Center, Tufts Medical Center, Boston, MA, USA; Department of Cardiology, University Medical Center Hamburg-Eppendorf, Martinistrasse 52, 20251 Hamburg, Germany; German Center for Cardiovascular Research (DZHK), Partner Site Hamburg/Luebeck/Kiel, Germany; Department of Cardiology, University Medical Center Hamburg-Eppendorf, Martinistrasse 52, 20251 Hamburg, Germany; German Center for Cardiovascular Research (DZHK), Partner Site Hamburg/Luebeck/Kiel, Germany; Department of Cardiology, University Medical Center Hamburg-Eppendorf, Martinistrasse 52, 20251 Hamburg, Germany; German Center for Cardiovascular Research (DZHK), Partner Site Hamburg/Luebeck/Kiel, Germany; Department of Cardiology, University Medical Center Hamburg-Eppendorf, Martinistrasse 52, 20251 Hamburg, Germany; German Center for Cardiovascular Research (DZHK), Partner Site Hamburg/Luebeck/Kiel, Germany; Department of Clinical Science and Education, Södersjukhuset, Karolinska Institutet, Sjukhusbacken 10, 11883 Stockholm, Sweden; Department of Cardiology, University Medical Center Hamburg-Eppendorf, Martinistrasse 52, 20251 Hamburg, Germany; German Center for Cardiovascular Research (DZHK), Partner Site Hamburg/Luebeck/Kiel, Germany; Institute of Cardiovascular Sciences, University of Birmingham, Edgbaston, Birmingham, B152TT, UK; Department of Cardiology, University Medical Center Hamburg-Eppendorf, Martinistrasse 52, 20251 Hamburg, Germany; German Center for Cardiovascular Research (DZHK), Partner Site Hamburg/Luebeck/Kiel, Germany; Center for Population Health Innovation (POINT), University Heart & Vascular Center Hamburg, University Medical Center Hamburg-Eppendorf, Hamburg, Germany; Department of Cardiology, University Medical Center Hamburg-Eppendorf, Martinistrasse 52, 20251 Hamburg, Germany; German Center for Cardiovascular Research (DZHK), Partner Site Hamburg/Luebeck/Kiel, Germany; Department of Cardiology, University Medical Center Hamburg-Eppendorf, Martinistrasse 52, 20251 Hamburg, Germany; German Center for Cardiovascular Research (DZHK), Partner Site Hamburg/Luebeck/Kiel, Germany; Department of Cardiology, University Medical Center Hamburg-Eppendorf, Martinistrasse 52, 20251 Hamburg, Germany; German Center for Cardiovascular Research (DZHK), Partner Site Hamburg/Luebeck/Kiel, Germany; Department of Clinical Science and Education, Södersjukhuset, Karolinska Institutet, Sjukhusbacken 10, 11883 Stockholm, Sweden

**Keywords:** Heart failure, Atrial fibrillation, Rhythm control, Outcomes, Mortality

Atrial fibrillation (AF) is common in patients with acute decompensated heart failure (HF). Both conditions share risk factors, e.g. hypertension, diabetes, and coronary artery disease, and aggravate each other.^1^ AF increases the risk of stroke, HF hospitalization, and mortality in acute decompensated HF.^2^ Rhythm control in this population remains challenging due to frequent recurrences and limited therapeutic options.

Early rhythm control reduces a composite outcome of cardiovascular death, stroke, or HF hospitalization by 21% compared with usual care in patients with AF and comorbidities, with consistent results in patients with AF and HF.^3,4^ While recent analyses indicate potential early benefits of rhythm control, the last randomized trial testing rhythm control in acute decompensated HF, ANDROMEDA, was halted nearly 20 years ago due to adverse safety signals.^5,6^

These findings underscore the clinical challenges of AF in acute decompensated HF. This study (i) characterized the profile of consecutive patients with AF and acute decompensated HF; (ii) examined associations between early rhythm control and all-cause mortality; and (iii) identified factors associated with the use and success of early rhythm control across the ejection fraction (EF) spectrum.

This retrospective analysis used data from the prospective CYCLE cohort (Characterisation of phenotYpes in aCute heart faiLure patiEnts) at the University Heart & Vascular Center Hamburg, Germany, described previously.^7^ The cohort enrolled consecutive patients hospitalized with acute decompensated HF defined by NT-proBNP ≥ 300 pg/mL and recent worsening of HF symptoms. Early rhythm control was defined as electrical or pharmacological cardioversion, antiarrhythmic drug therapy, or catheter ablation during the index hospitalization. Success of early rhythm control was defined as sinus rhythm at discharge. The primary objective was to evaluate the association between early rhythm control and all-cause mortality. To detect potential confounders, baseline characteristics were compared between patients with and without rhythm control, and factors associated with early rhythm control were determined. Continuous and categorical variables were analysed using appropriate non-parametric and chi-square tests, multivariable logistic and Cox regression models adjusted for age, sex, and lactate >2.2 mmol/L were applied to identify predictors and associations with all-cause mortality.

In 141 patients with AF and acute decompensated HF, 72 (51.1%) received early rhythm control. Patients treated with early rhythm control were younger (75 vs. 79 years), more often had new-onset HF (36.9% vs. 14.1%), and showed lower rates of anemia (36.6% vs. 63.8%), chronic kidney disease (26.4% vs. 45.6%), ischemic cardiomyopathy (23.6% vs. 45.5%), diabetes (21.1% vs. 40.6%), and higher rates of HFrEF (76.2% vs. 46.6%) than patients without rhythm control. Over a median follow-up of 2.97 (95% CI 2.49–3.47) years, use of early rhythm control was associated with a lower risk of all-cause mortality (adjusted HR 0.46, 95% CI 0.26–0.82; *P* = 0.009) (*Figure 1A*, *Figure 1C*). Successful early rhythm control was associated with a lower risk of all-cause mortality (adjusted HR 0.38, 95% CI 0.19–0.75; *P* = 0.006) (*Figure 1B*, *Figure 1C*). In multivariable analysis, early rhythm control was independently associated with new-onset HF. Lower use of early rhythm control was independently associated with beta-blocker and loop diuretic therapy, and with anemia and coronary artery disease (*Figure 1D*). Successful early rhythm control was less likely in older patients and in those with hypertension, beta-blocker therapy, or loop diuretic use (*Figure 1E*).

**Figure 1:**
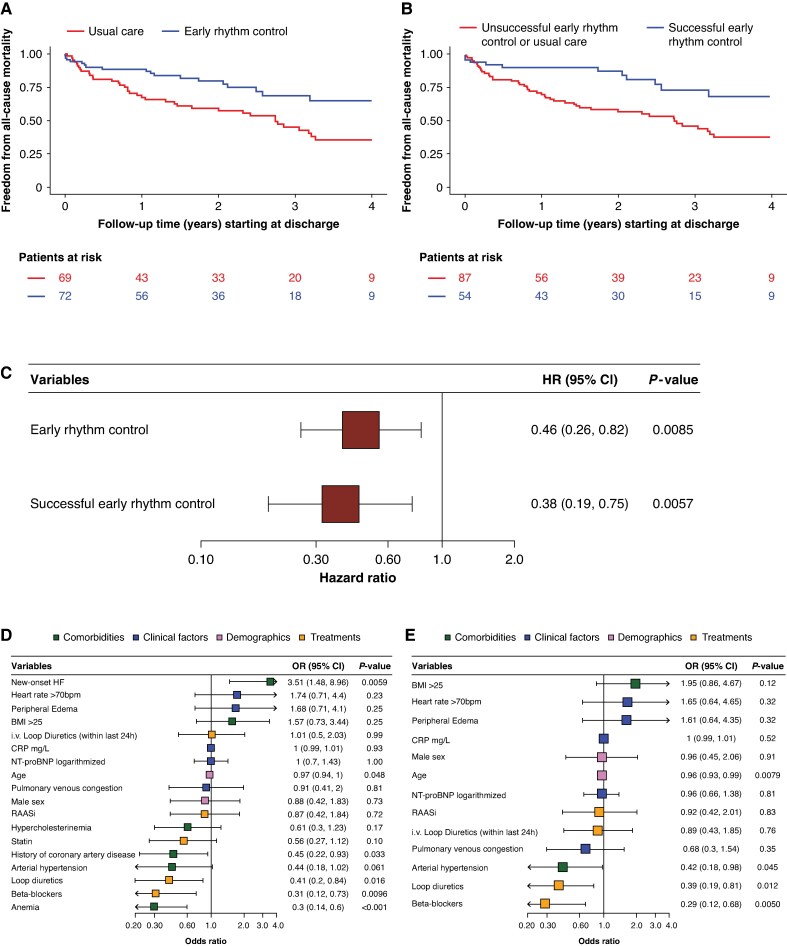
Associations of use of early rhythm control (*A*) and successful early rhythm control (*B*) with all-cause mortality. Kaplan–Meier estimates were used to illustrate cumulative all-cause mortality over time. Multivariable Cox regression analyses of early rhythm control and successful early rhythm control associated with all-cause mortality, adjusted for age, sex, and lactate levels >2.2 mmol/L. Results are shown as adjusted HRs with 95% CI and illustrated in a forest plot (*C*). Multivariable logistic regression analyses identifying associations between clinical factors and (*D*) the use of early rhythm control and (*E*) successful rhythm control, defined as sinus rhythm at discharge. Results are shown as adjusted ORs with 95% CI and illustrated in a forest plot. Abbreviations: BMI, body mass index; CI, confidence interval; OR, odds ratio; CRP, C-reactive protein; HF, heart failure; HR, hazard ratio; i.v., intravenously; NT-proBNP, N-terminal pro-B-type natriuretic peptide; RAASi, renin–angiotensin–aldosterone system inhibitors.

Overall, these findings indicate that early rhythm control was initiated in about ∼50% of all cases, illustrating clinical uncertainty. Early rhythm control and successful early rhythm control were associated with better survival over a 3-year follow-up. This analysis cannot differentiate whether this is due to patient selection or a beneficial effect of therapy: Patients who received early rhythm control generally presented with fewer comorbidities and predominantly with HFrEF, reflecting clinical practice patterns observed in EAST-AFNET 4 and AF-CHF.^3^ These findings suggest that physicians tend to prioritize rhythm control in clinically stable or newly diagnosed cases. Successful rhythm restoration was more frequent in HFrEF, potentially due to less atrial fibrosis and structural remodelling, in line with mechanistic insights from the DECAAF study.^8^ In line with results from CASTLE-AF and CASTLE-HTx, our data indicate that rhythm control may be both feasible and prognostically relevant across the spectrum of HF severity.^9,10^

Furthermore, early rhythm control use was independently associated with improved survival, and the greatest benefit was seen in patients discharged in sinus rhythm, supporting the importance of rhythm restoration itself. These hypothesis-generating findings are in line with results in the EAST-AFNET 4 HF subanalysis and with CASTLE-AF and CASTLE-HTx and suggest beneficial effects of early rhythm control in patients with acute decompensated HF, a group largely excluded from prior trials. External validation and testing in a controlled clinical trial are needed.

This was a single-centre, retrospective analysis subject to residual confounding, selection bias, and lack of randomization. Treatment decisions reflected local procedures and physician preferences. The cohort was predominantly of European origin, which may limit generalizability. Successful rhythm control was defined as sinus rhythm at discharge without long-term rhythm or symptom assessment, and data on quality of life were unavailable. The modest sample size further restricted subgroup analyses.

In conclusion, in patients hospitalized with AF and acute decompensated HF, early rhythm control was initiated in ∼50% of cases and achieved sinus rhythm at discharge in ∼75% of treated patients. Use of early rhythm control was more likely in younger individuals with new-onset HF and fewer comorbidities, and less common in those with anemia and history of coronary artery disease or receiving diuretics or beta-blockers. Both initiation and success of early rhythm control were independently associated with lower all-cause mortality, suggesting a potential prognostic benefit that warrants confirmation in randomized trials.

## Data Availability

Data are available on reasonable request.
